# Assembly of the Type Two Secretion System in *Aeromonas hydrophila* Involves Direct Interaction between the Periplasmic Domains of the Assembly Factor ExeB and the Secretin ExeD

**DOI:** 10.1371/journal.pone.0102038

**Published:** 2014-07-15

**Authors:** Elizabeth M. Vanderlinde, Su Zhong, Gang Li, Dariusz Martynowski, Pawel Grochulski, S. Peter Howard

**Affiliations:** 1 Department of Microbiology and Immunology, University of Saskatchewan, Saskatoon, Saskatchewan, Canada; 2 Canadian Light Source, Saskatoon, Saskatchewan, Canada; 3 College of Pharmacy and Nutrition, University of Saskatchewan, Saskatoon, Saskatchewan, Canada; Centre National de la Recherche Scientifique, Aix-Marseille Université, France

## Abstract

The type two secretion system is a large, trans-envelope apparatus that secretes toxins across the outer membrane of many Gram-negative bacteria. In *Aeromonas hydrophila*, ExeA interacts with peptidoglycan and forms a heteromultimeric complex with ExeB that is required for assembly of the ExeD secretin of the secretion system in the outer membrane. While the peptidoglycan-ExeAB (PG-AB) complex is required for ExeD assembly, the assembly mechanism remains unresolved. We analyzed protein-protein interactions to address the hypothesis that ExeD assembly in the outer membrane requires direct interaction with the PG-AB complex. Yeast and bacterial two hybrid analyses demonstrated an interaction between the periplasmic domains of ExeB and ExeD. Two-codon insertion mutagenesis of *exeD* disrupted lipase secretion, and immunoblotting of whole cells demonstrated significantly reduced secretin in mutant cells. Mapping of the two-codon insertions and deletion analysis showed that the ExeB-ExeD interaction involves the N0 and N1 subdomains of ExeD. Rotational anisotropy using the purified periplasmic domains of ExeB and ExeD determined that the apparent dissociation constant of the interaction is 1.19±0.16 µM. These results contribute important support for a putative mechanism by which the PG-AB complex facilitates assembly of ExeD through direct interaction between ExeB and ExeD. Furthermore, our results provide novel insight into the assembly function of ExeB that may contribute to elucidating the role of homologous proteins in secretion of toxins from other Gram negative pathogens.

## Introduction

The secretion of enzymes and exotoxins by the type two secretion system (T2SS) is a widespread virulence mechanism of Gram negative bacterial pathogens [1]–[4]. The T2SS is a large, trans-envelope apparatus composed of 12–16 proteins designated GspC-O, which are highly conserved, and GspAB and GspS, which are variably found among different bacterial species. The basic structure of the T2SS includes an inner membrane platform (GspC, GspE, GspF, GspL, GspM), a periplasmic pseudopilus (GspG-K), and an outer membrane channel formed by the secretin protein GspD. Substrates of the T2SS are translocated from the cytosol to the periplasmic space by the Sec or Tat pathways, and folded proteins are then exported across the outer membrane through the pore formed by GspD [5]–[9].

GspD is an 80 kDa protein with an N-terminal periplasmic domain and a C-terminal outer membrane domain that forms multimers in the outer membrane [10]. Electron microscopy studies determined that the secretin forms a dodecamer with a central pore of 50–100 Å. [5], [10], [11]. Crystal structures of the periplasmic domain of the GspD homologues from enterotoxigenic *Escherichia coli* (ETEC), and *Pseudomonas aeruginosa* have been described [13]–[15]. The structural data revealed that it is composed of four subdomains, denoted N0-N3 [13]. In addition, recent structural studies by Van der Meeren *et al*. have shown that GspD monomers in *P. aeruginosa* form dimers prior to assembly into the dodecameric secretin [14]. The crystal structure of the C-terminal domain of GspD has not been solved to date, however cryo-electron microscopy data was used to reconstruct the secretin from *Vibrio cholerae* at a resolution of 19 Å. The secretin formed a dodecameric barrel-like structure, with the C-terminal forming the transmembrane channel and the periplasmic domain forming a vestibule that is proposed to interact with the substrate prior to translocation across the outer membrane. The diameter of the periplasmic gate in the closed state was found to be ∼55 Å suggesting that substantial structural changes must occur to accommodate large substrates such as cholera toxin, which has a diameter of ∼65 Å [12].

Assembly of the secretin in *Aeromonas hydrophila* and *A. salmonicida* requires an inner membrane protein complex composed of ExeA, a 60 kDa AAA ATPase, and ExeB, a basic 25 kDa protein [16]–[18]. In the absence of the ExeAB complex, ExeD monomers remain in the inner membrane [16]. Over-expression of ExeD in an ExeAB mutant suppressed the secretion defect by enabling assembly of some ExeD multimer in the outer membrane. Sucrose density gradient centrifugation and immunoblotting determined that over-expression of ExeD in an ExeAB mutant led to accumulation of multimer in both membranes, with the majority of the assembled ExeD remaining in the inner membrane, whereas in a wild-type background most of the assembled ExeD was found in the outer membrane [16].

ExeA and ExeB both have a single transmembrane domain and large periplasmic domains [19], [20]. ExeA has a cytoplasmic ATPase domain that is required for complex formation with ExeB and secretin multimerization [17]. The periplasmic domain of ExeA contains a single peptidoglycan binding domain that has been shown to bind peptidoglycan both *in vivo* and *in vitro* [21]–[24]. Interaction with peptidoglycan resulted in multimerization of ExeA and ExeB into large heteromultimeric complexes of up to 12 monomers of each protein [22]. Mutation of several conserved amino acids in the peptidoglycan binding domain of ExeA has also shown that the interaction with peptidoglycan is required for assembly of ExeD [23]. These results suggest that the ExeAB complex may be chaperoning the secretin through the cell wall, or may be required for assembly of the secretin within both the peptidoglycan layer and the outer membrane, as suggested by the size of this and other secretins that appear to completely cross the periplasm [1], [25]. Like ExeA, ExeB is absolutely required for assembly of ExeD, however, ExeB has no known functional domains and its role in secretin assembly is unknown [19], [20].

While the peptidoglycan-ExeAB (PG-ExeAB) complex is required for multimerization of ExeD, the precise role of the complex in the transport or assembly of ExeD is unknown. In this study we analyzed protein-protein interactions to address the hypothesis that the ExeD secretin is recruited for assembly into the peptidoglycan and/or the outer membrane by direct interaction with the ExeAB complex. We used yeast two hybrid and bacterial two hybrid analyses to identify and quantify an interaction between ExeD and ExeB. Two-codon insertion mutagenesis and deletion analysis determined that the interaction requires the N0 and/or N1 subdomains of ExeD. The results were further confirmed by co-purification, and the dissociation constant for the interaction was determined by purification of the periplasmic domains of ExeB and ExeD followed by fluorescence anisotropy. These results support a direct role for the peptidoglycan-ExeAB complex in ExeD assembly and suggest that the role of ExeB is to act as a scaffold for assembly by interacting with both ExeA and ExeD.

## Materials and Methods

### Strains, media, and growth conditions

The strains and plasmids used are summarized in Table 1. *Saccharomyces cerevisiae* haploid strain PJ69-4A was grown at 30°C in complete yeast extract peptone-dextrose (YPD) medium or synthetic dextrose (SD) medium supplemented as necessary with Trp, Leu, and His [26]. *A. hydrophila* strains were grown at 30°C in buffered Luria Bertani (LB) medium [16]. *E. coli* strains were cultured in 2× YT (16 g tryptone, 10 g yeast extract, and 5 g NaCl per litre) at 37°C. Antibiotics were used at the following final concentrations when necessary (µg•mL^−1^): ampicillin (Ap), 100; chloramphenicol (Cm), 2.5; kanamycin (Km), 50; tetracycline (Tc), 10.

**Table 1:** List of strains, plasmids, and PCR fragments used.

Strain	Description	Reference
*A. hydrophila*		
Ah65	Wild type	This laboratory
AhD14	Ah65 Δ*exeD*	[16]
C5.84	Ah65 *exeA*::Tn5-751	[18]
*E. coli*		
XL-1 Blue	Cloning host; Tc^r^	Stratagene
S17-1	Conjugation donor; St^r^	[40]
BL21(DE3)	Expression host	Novagen
BM2H	Bacterial two-hybrid host	Agilent Technologies
*S. cerevisiae*		
pJ69-4A	Yeast two-hybrid host	[26]
*Plasmids*		
pBluescript II SK+	Cloning vector; *lac* promoter; Ap^r^	Stratagene
pCDFDuet-1	Expression vector; T7*lac* promoter; Sm^r^	Novagen
pET30a	Expression vector; T7lac promoter; Km^r^	Novagen
pGBT9	Gal4_BD_ fusion vector; ADH1 promoter; Ap^r^	Clontech
pGAD424	Gal4_AD_ fusion vector; ADH1 promoter; Ap^r^	Clontech
pBT	Lambda cI fusion vector; *lacZ* and HIS3 promoters; Cm^r^	Agilent Technologies
pTRG	RNA polymerase fusion vector; *lacZ* and HIS3 promoters; Tc^r^	Agilent Technologies
pMMB207	Wide host range vector; *tac* promoter; Cm^r^	[41]
pPH 14.5	*exeCD* BglII in *Bam*HI of pBluescript II SK+	[16]
pVACD-P	promoter-less *exeCD* in XbaI/Hind III of pMMB207	[16]
MUS81	Yeast two-hybrid positive control; Ap^r^	[27]
MMS4	Yeast two-hybrid positive control; Ap^r^	[27]
pTRG-Gal11P	Bacterial two-hybrid positive control encoding a domain (90aa) of the mutant form of the Gal11 protein; Tc^r^	Agilent Technologies
pBT-LGF2	Bacterial two-hybrid positive control encoding the dimerization domain (40 aa) of the Gal4 transcriptional activator protein; Cm^r^	Agilent Technologies
pVA59**	pVACD-P containing linker insertion at aa 59	This study
pVA128**	pVACD-P containing linker insertion at aa 128	This study
pVA138**	pVACD-P containing linker insertion at aa 138	This study
pVA183**	pVACD-P containing linker insertion at aa 183	This study
pVA203**	pVACD-P containing linker insertion at aa 203	This study
pVA248**	pVACD-P containing linker insertion at aa 248	This study
pVA257**	pVACD-P containing linker insertion at aa 257	This study
pVA270**	pVACD-P containing linker insertion at aa 270	This study
*Gene fragments* *		
P-*exeA*	Cloned into pGBT9 and pGAD424	This study
P-*exeB*	Cloned into pGBT9, pGAD424, pBT, pTRG, and pET30a	This study
P-*exeC*	Cloned into pGBT9 and pGAD424	This study
P-*exeD*	Cloned into pGBT9, pGAD424, pBT, pTRG, pET30a and pCDFDuet	This study
P-*exeDN0*	Cloned into pGBT9, pGAD424, pBT, pTRG, pET30a and pCDFDuet	This study
P-*exeDN0N1*	Cloned into pGBT9, pGAD424, pBT, pTRG, pET30a and pCDFDuet	This study
P-*exeDN1N2N3*	Cloned into pGBT9, pGAD424, pBT, pTRG, pET30a and pCDFDuet	This study
P-*exeDN2N3*	Cloned into pGBT9, pGAD424, pBT, pTRG, pET30a and pCDFDuet	This study
P-*exeL*	Cloned into pGBT9 and pGAD424	This study
P-*exeM*	Cloned into pGBT9 and pGAD424	This study
P-*exeN*	Cloned into pGBT9 and pGAD424	This study

*Refer to materials and methods for details regarding cloning of PCR products for yeast two-hybrid, bacterial two-hybrid, and co-purification analyses.

**Fragments of P-ExeD containing two codon insertion mutations cloned into pGBT9 and pGAD424 for yeast two-hybrid analysis, as described in the materials and methods.

### Plasmid construction

Primers used are listed in Table 2. DNA sequences encoding the periplasmic domains of ExeA, ExeB, ExeC, ExeD, ExeL, ExeM, and ExeN were amplified from the *A. hydrophila* Ah65 chromosome and cloned into *Eco*RI and *Bam*HI restriction sites in pGBT9 and pGAD424 (Clontech), creating fusion proteins to GAL4_BD_ and GAL4_AD_, respectively. The same strategy was also used to construct fusions containing different subdomains of P-ExeD. Constructs for yeast two-hybrid analysis of two-codon insertion mutations were made by PCR amplifying the pVA linker-insertion plasmids, and cloning them into pGBT9-P-ExeD (EcoRI and MscI) or pGAD424-P-ExeD (EcoRI and AarI).

**Table 2:** List of primers.

Name	Oligonucleotides (5′-3′)		
	Yeast two-hybrid	Bacterial two-hybrid	Co-purification
P-*exeA*	ATCCAGAATTCCAGTTCTTCGGCTTCTTCCC	na	na
	ATACTGGATCCTCAGGAAGCCTCCTCCGAC	na	na
P-*exeB*	TTAAAGAATTCAACCGCCCCATCGAGAAGAC	GATATAGCGGCCGCAAACCGCCCCATCGAGAAGAC	CATATGCACCATCATCATCATCATAACAACCGCCCCATCGAGAAG
	TTAATGGATCCTCAGCCGCGCCAGTCCTG	TTAATGCTCGAGTCAGCCGCGCCAGTCCTG	CTCGAGAAGGGGAGCTCTCAGGCTCC
P-*exeC*	TTAAAGAATTCCGTCTGCTGGATCTCGGC	na	na
	TTAAAGGATCCTTATTCTGACAAGCCGACATAAAC	na	na
P-*exeD*	TTAATGAATTCACCGAGTATTCTGCCAGCTTC	GATATAGCGGCCGCAACCGAGTATTCTGCCAGCTT	TTAATCATATGACCGAGTATTCTGCCAGCTTC
	TTAATGGATCCTCACAGTACCTGGGCGCGG	TTAATGCTCGAGTCACAGTACCTGGGCGCGG	TTAATCTCGAGCAGTACCTGGGCGCGG
P-*exeD N0*	TTAATGAATTCACCGAGTATTCTGCCAGCTTC	TTAATGCTCGAGTCACAGTACCTGGGCGCGG	ATCCACATATGCAGTTCTTCG
	TTAATGGATCCTTAGCCCGGGTTGGTCTCAT	TTAATGCTCGAGTCACAGTACCTGGGCGCGG	TTAATGCTCGAGTTAGCCCGGGTTGGTCTCAT
P-*exeD N0N1*	TTAATGAATCCACCGAGTATTCTGCCAGCTTC	TTAATGCTCGAGTCACAGTACCTGGGCGCGG	ATCCACATATGCAGTTCTTCG
	ATAATGGATCCTTAGGCATATTTCAGCTTGATGAT	TTAATGCTCGAGTCACAGTACCTGGGCGCGG	TAATGCTCGAGTTAGGCATATTTCAGCTTGATGAT
P-*exeD N1N2N3*	TAGCTGAATTCATAGGCGACGAGATGGTGACC	GATATAGCGGCCGCAATAGGCGACGAGATGGTGACC	GATATACATATGATAGGCGACGAGATGGTGACC
	TTAATGGATCCTCACAGTACCTGGGCGCGG	GATATAGCGGCCGCAACCGAGTATTCTGCCAGCTT	TTAATCTCGAGCAGTACCTGGGCGCGG
P-*exeD N2N3*	AATATGAATTCTCCGCCGGCGAGATGGT	GATATAGCGGCCGCATCCGCCGGCGAGATGGT	GATATACATATGTCCGCCGGCGAGATGGT
	TTAATGGATCCTCACAGTACCTGGGCGCGG	GATATAGCGGCCGCAACCGAGTATTCTGCCAGCTT	TTAATCTCGAGCAGTACCTGGGCGCGG

Bacterial two-hybrid plasmids were constructed by amplifying the DNA sequence encoding the periplasmic domains of ExeB and ExeD from pGAD424-P-ExeB, and pGAD424-P-ExeD, respectively. PCR products were cloned into NotI and XhoI restriction sites in either the bait plasmid pBT or the target plasmid pTRG (Agilent Technologies). All constructs were confirmed by restriction digest analysis and sequencing. Primers used in the study are listed in Table 2.

The periplasmic domain of ExeB was PCR amplified from pRJ31.1 and ligated into the NdeI and XhoI sites of pET30a (Novagen) to construct pNHis-P-ExeB. The plasmid pP-ExeD was constructed by amplifying the P-ExeD fragment from pBD-P-ExeD and ligating into the NdeI and XhoI restriction sites of pCDFDuet-1 (Novagen). A similar approach was also used to make the constructs containing different combinations of the N0, N1, N2, and N3 domains of ExeD. The pNHis-P-ExeD plasmid for purification of P-ExeD was made by cloning the P-ExeD fragment from pBD-P-ExeD into the NdeI and XhoI sites of pET30a.

### Yeast two-hybrid analysis

The yeast strain PJ69-4A was co-transformed with different combinations of pGBT9 (GAL_BD_) and pGAD424 (GAL_AD_) protein fusions. Co-transformants were selected on SD-Trp-Leu. At least 5 independent colonies per co-transformant were grown up in SD-Trp-Leu, plated on SD-Trp-Leu, and SD-Trp-Leu-His. Activation of the P*_GAL1_-HIS3* reporter was assessed after incubation at 30°C for 3 d. The fusion proteins MUS81 and MMS4 were used as a positive control [27].

### Two-codon insertion mutagenesis

Two-codon linker insertion mutagenesis of *exeCD* encoded in pPH 14.5 was performed as described previously [16], [22], except that GATCCG and CGGATC were used as linkers to create a unique BamHI site. A 1.3 kb BamHI fragment encoding Km resistance derived from pUC-4K was ligated with the BamHI-linearized linker-pPH 14.5 plasmids to facilitate selection of linker-containing plasmids. The Km^r^ cassette was removed by BamHI digestion followed by ligation and electroporation into XL-Blue cells. Eleven insertions in ExeD were isolated. Eight were mapped to the periplasmic domain of ExeD and used in this study (see Table 3 for the locations). The insertion mutations were moved to pVACD-P [16] by replacement of appropriate restriction fragments of CalI/AatII, AatI/MreI, or MreI/HindIII for *in vivo* analysis. The insertions were confirmed by DNA sequencing.

**Table 3:** Yeast two hybrid analysis of P-ExeB and P-ExeD two codon insertion mutants (D_m_)*.

	Two codon insertions							
Insertion site	59	128	138	183	203	248	257	270
Subdomain	N0	N1	N1	N1	N2	N2	N2	N3
Residues inserted	IR	IR	IR	IR	SG	DP	IR	IR
B-D_m_	−	+	+	+/−	+	+	+	+
D_m_-B	−	−	−	−	+	+	+	+
D-D_m_	+	+	−	−	−	−	−	+/−
D_m_-D	+	−	−	−	−	−	−	+/−

*Interactions were assayed as described in Figure 2. (+): positive; (−): negative; (+/−): weak positive.

### Protein analysis

SDS-PAGE gels (12%) were routinely used to analyze protein samples. For analysis of ExeD secretin, 3–8% Criterion pre-cast polyacrylamide Tris-acetate gradient gels (Biorad) were used. Gradient gel samples were standardized to 0.01 OD_600_ per lane. For immunoblotting, the proteins were transferred to PVDF membranes (GE Healthcare Life Sciences). Visualization of ExeD was achieved by incubation with the appropriate rabbit antiserum followed by incubation with peroxidase-conjugated mouse anti-rabbit IgG (Sigma). The signal was developed with a chemiluminescent substrate kit (GE Healthcare Life Sciences).

### Lipase secretion assay

Lipase activity was assayed by measuring the increase in absorbance at 410 nm from the release of *p*-nitrophenol from *p*-nitrophenol caprylate (pNPC) as described previously [28]. The culture supernatants (200 µL) were added to 800 µL substrate buffer containing 1 mM *p*-nitrophenol caprylate, 100 mM Tris pH 8.0, and 0.2% Triton-X 100, and the reaction was incubated for 30 min at RT, during which the OD was measured at five min intervals. One unit of lipase activity equals 1 nmol pNPC hydrolyzed per min. The lipase activity per mL supernatant per OD_600_ of culture was compared to that of the wild type strain to calculate the percentage of lipase secretion.

### Bacterial two-hybrid analysis

The BacterioMatch two-hybrid system reporter strain (BM2H) was co-transformed with different combinations of the bait (pBT) and target (pTRG) plasmids containing the *exeB* and *exeD* fusions. Co-transformants were selected on LB with chloramphenicol, tetracycline, and kanamycin and confirmed with PCR.

β-galactosidase activity of the co-transformants was measured using a modification of the assay described by Slauch and Silhavy [29]. Briefly, co-transformants were sub-cultured 1∶125 in 10 mL of LB,Cm,Tc,Km with 0.02 mM IPTG to an OD_600_ of 2.0. Permeabilized cell suspensions were prepared by vortexing a 1 mL aliquot of cells with 10 µL 0.1% SDS and 20 µL chloroform for 30 s, followed by a 5 min incubation at RT. To measure β-galactosidase activity, 100 µL of cell suspension was added to 900 µL of reaction solution (900 µL Z-buffer, 10 mg•mL^−1^ ONPG), and the absorbance at 420 nm was measured at 5 min intervals for 20 min in an Ultrospec 3000 spectrophotometer (GE Healthcare Life Sciences). All assays were performed in triplicate and at least 3 independent experiments were performed for each co-transformant.

Minimum inhibitory concentration assays for carbenicillin were performed by sub-culturing the co-transformants 1∶100 in 1 mL of LB,Cm,Tc,Km with 0.1 mM IPTG and serial dilutions of carbenicillin (0–12.8 mg•mL^−1^). Results were determined after overnight incubation at 30°C with shaking. At least three independent replicates were performed for each co-transformant.

### Co-purification analysis of ExeB-ExeD interactions

*E. coli* strain BL21(DE3) was sequentially co-transformed with pN-His-P-ExeB and pP-ExeD, pP-ExeDN0, pP-ExeDN0N1, pP-ExeDN1N2N3, or pP-ExeDN2N3. Co-transformants were grown in 2×YT and induced with 1 mM IPTG for 4.5 h. Cell lysates were prepared and applied to a HisTrap HP 1 mL column (GE Healthcare Life Sciences) as described previously [21], except that 50 mM Tris-HCl, 50 mM NaCl, 1 mM phenylmethylsulphonyl fluoride (PMSF), pH 7.5 was used as the binding buffer. The column was washed with binding buffer and eluted with 100% elution buffer (50 mM Tris-HCl, 50 mM NaCl, 1 mM PMSF, 500 mM imidazol, pH 7.5). The elution fractions were analyzed by SDS-PAGE stained with coomassie brilliant blue or immunoblotted with αExeD serum.

### Steady state rotational anisotropy assays

The binding affinity between the periplasmic domains of ExeB and ExeD was measured by steady state fluorescence depolarization (rotational anisotropy), as described by Feng *et al*. [30]. The genes encoding the periplasmic domains of *exeB* and *exeD* were cloned into the expression vector pET30a and the N-His-tagged-P-ExeB and N-His-tagged-P-ExeD protein fragments were over-expressed in *E. coli* BL21. Lysates from BL21 were applied to a Ni-NTA column and the His-tagged proteins were eluted with a 0–500 mM gradient of imidazole. The eluted fractions were desalted into ion exchange buffer and further purified with a Resource S ion exchange column. The Fluorescein-EX Protein Labeling kit (Invitrogen) was used to label P-ExeB with fluorescein. The degree of labeling was determined by gel chromatography to be ∼0.8 moles of dye per mole of protein. The reaction mixture (50 µL) contained F-labeled P-ExeB (50 nM), RT buffer, and a titration of ExeD (0–4000 nM). Data collection and anisotropy calculations were performed at 21°C on a QuantaMaster QM-4 spectrofluorometer (Photon Technology International) with a dual emission channel. Samples were excited with vertically polarized light at 495 nm (6-nm band pass) and vertical and horizontal emissions were measured at 520 nm (6-nm band pass). The apparent dissociation constant (K_d_) was calculated by fitting data to a rectangular hyperbola using SigmaPlot 11.2 software.

### Model building

Sequence homology analysis indicated that the highest similarity between the ExeD protein sequence and a protein of known structure is to the periplasmic N-terminal domain of GspD from enterotoxigenic *E. coli* (PDB; 3EZJ) [13]. The sequence identity for amino acids 7–239 is 59%, therefore this molecule was used to create a model of the periplasmic domain of ExeD from amino acids 26 to 258. Since there was no available structure of the fragment from 259 to 305 only secondary structure assignment was used for these residues representing mostly the N3 subdomain.

## Results

### Yeast two-hybrid analysis of interactions between the periplasmic domain of ExeD and other components of the type two secretion system

**Figure 1:**
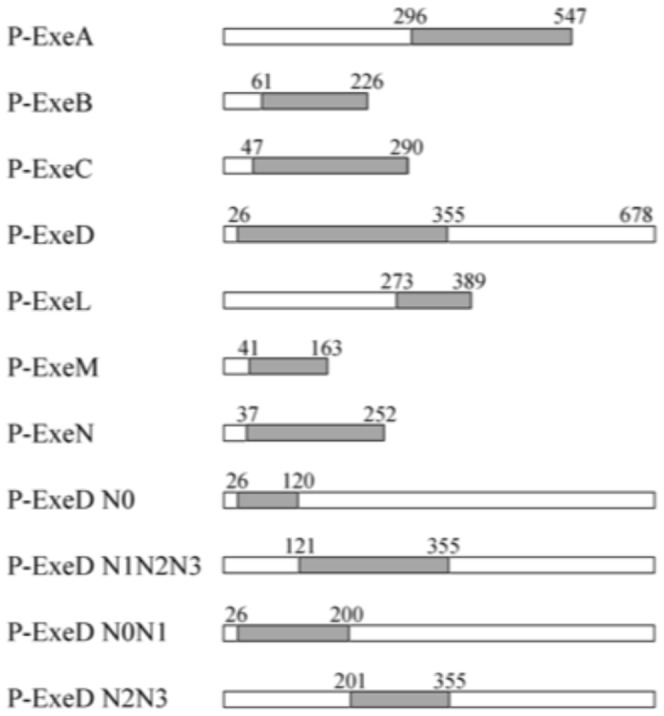
Exe protein derivatives used in this study. The grey boxes indicate periplasmic domains analyzed in the study. The ExeD deletion constructs containing different subdomains are also indicated. Residues bordering the domains are listed above the constructs.

**Figure 2:**
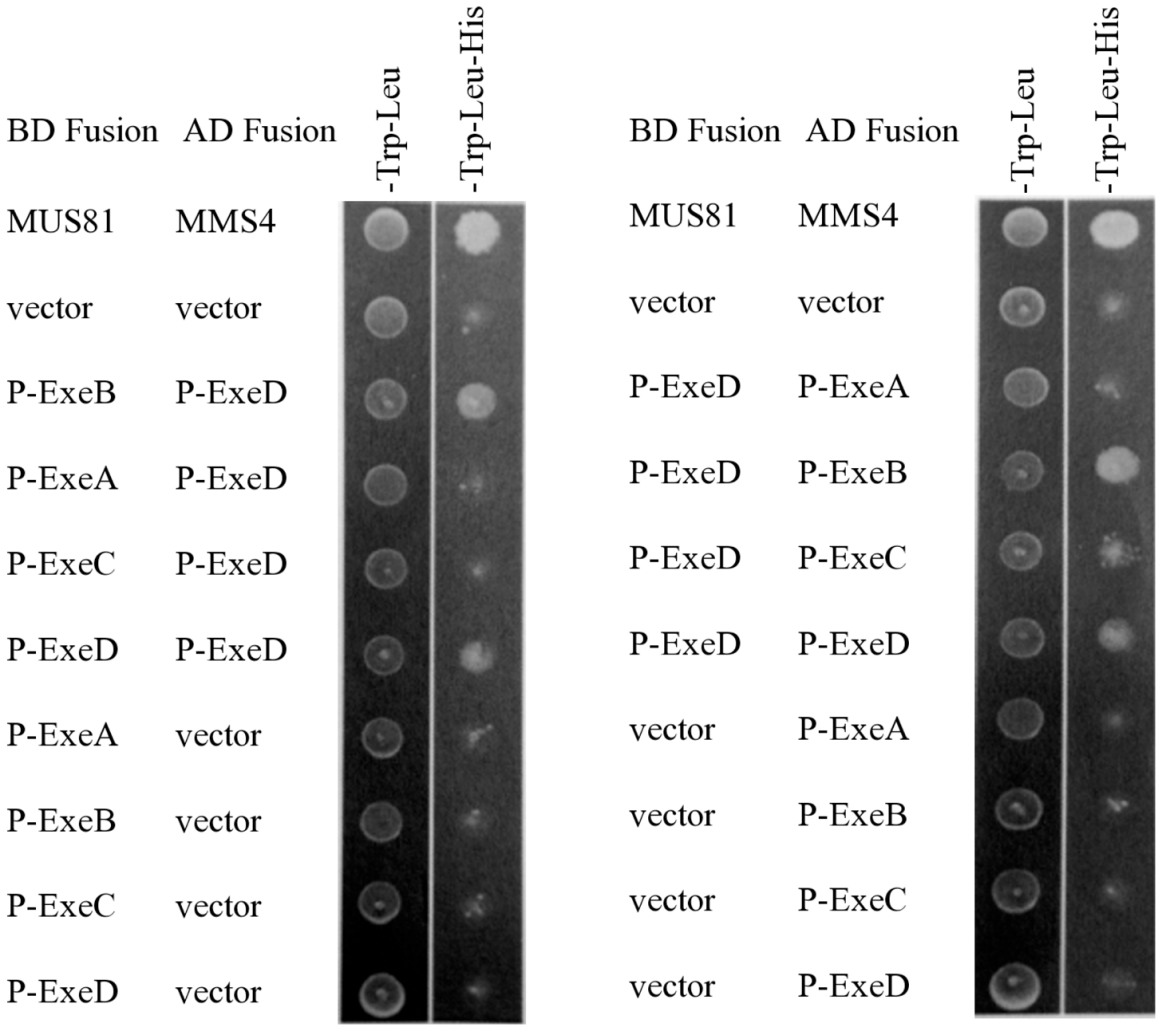
Interaction between ExeA, ExeB, ExeC, and ExeD by yeast two-hydrid analysis. The periplasmic domains of ExeA, B, C, and D were fused to Gal4 BD domain and assayed for interactions with the periplasmic domain of ExeD fused to the Gal4 AD domain (left panel). The interactions were also assayed in the reverse fusion orientation (right panel). MUS81 and MMS4 were included as the positive control [27]. Co-transformants were selected with -Trp-Leu medium and activation of the reporter gene *gal1-his3* was selected with -Trp-Leu-His medium.

We used a yeast two-hybrid system to analyze interactions between the periplasmic (P-) domain of ExeD and the periplasmic domains of ExeA, ExeB, ExeC, ExeD, ExeL, ExeM, and ExeN. Constructs used for yeast two-hybrid assays are shown in Fig. 1. Activation of the *gal1*-*his3* reporter gene was determined by growing co-transformants on minimal synthetic medium that lacked tryptophan, leucine, and histidine. There was no detectable interaction between ExeD and ExeA (Fig. 2), ExeD and ExeL, ExeD and ExeM, or ExeD and ExeN (data not shown). Interactions were identified between the following BD-AD fusions: P-ExeB and P-ExeD, P-ExeD and P-ExeB, P-ExeD and P-ExeD (Fig. 2). As well, the data indicate a possible weak interaction between P-ExeD and P-ExeC, however, the interaction was not observed in the opposite orientation (Fig. 2), which could be due to either the weakness of the interaction, or because the proteins were not in the correct orientation. The interaction between GspC and GspD has been reported previously for homologous proteins in *Vibrio cholera, Dickeya didantii*, and *Pseudomonas aeruginosa* [30]–[33], however, it was not identified in a previous yeast two hybrid study in *E. chrysthanthemum* (7). In addition, the strongest interaction we observed was between the periplasmic domains of ExeB and ExeD, and consequently this interaction was the focus of the remainder of this study.

### Two-codon insertion mutagenesis analysis of P-ExeD

A series of two-codon linker insertion mutations in full-length *exeD* were constructed to determine the regions that are critical for the interaction between ExeB and ExeD. A total of 11 mutants were isolated and the insertions mapped using the N0, N1, N2, and N3 subdomains of the N-terminus reported for the homologous secretin protein, GspD as reference points. [13]. Eight of the mutations were in the N0 [1], N1 [3], N2 [3], and N3 [1] N-terminal sub-domains (Table 3), while the other three insertions were located in the C-terminal portion of ExeD and were not analyzed further.

Yeast two-hybrid analysis was used to qualitatively determine how the two-codon insertions affected the interaction between P-ExeB and P-ExeD (Table 3). The periplasmic domains of the insertion mutants of *exe*D were cloned into pGBT9 and pGAD424, as described in the materials and methods. Interaction between P-ExeB and P-ExeD was disrupted by the insertion in the N0 subdomain, and partially affected by an insertion in the C-terminal portion of the N1 subdomain, whereas the P-ExeD and P-ExeB interaction was disrupted by all four of the insertions in the N0 and N1 subdomains. Similar experiments were conducted with P-ExeD-P-ExeD fusions, and the insertions that disrupted the interaction were found in the N1 and N2 subdomains. These data suggest that the two-codon insertions did not disrupt the over-all folding of P-ExeD, although they may have altered local folding.

**Figure 3:**
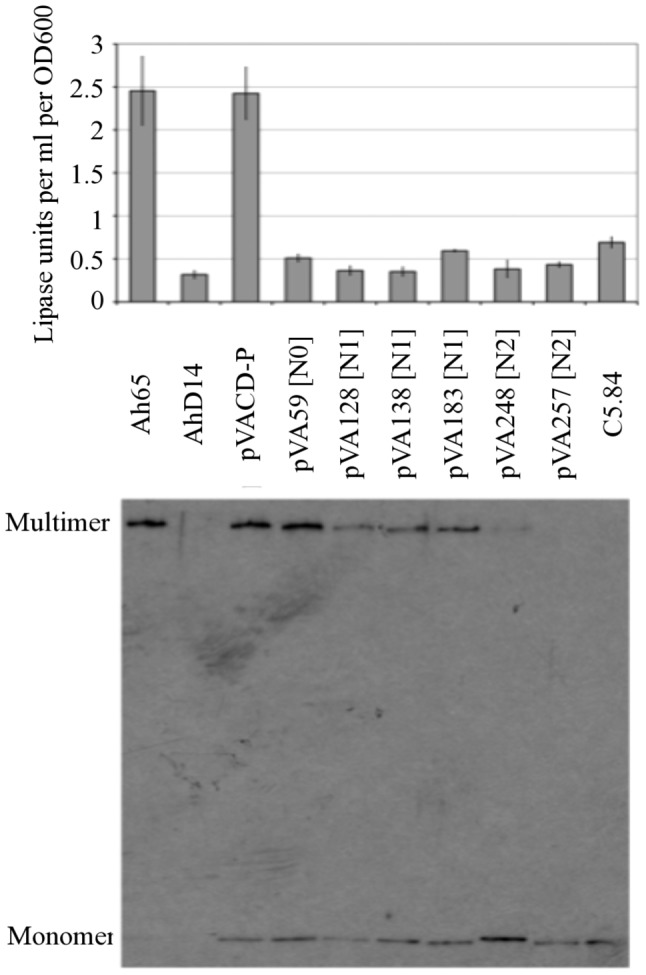
Lipase secretion (top) and secretin assembly (bottom) by the two codon insertion mutants. Data presented are the average lipase activity of three independent cell cultures compared to the wild-type strain. Error bars indicate the standard deviation. Assembly of the secretin was analyzed as described in the materials and methods. The *exeAB* ^−^ strain C5.84 was used as a negative control. The ExeD multimers and monomers are indicated.

The effect of the two-codon insertion mutations on *in vivo* secretion and secretin assembly was also determined (Fig. 3). Full length *exeD* with the two codon insertion mutations were expressed from the broad host range plasmid pMMB207 in the *exeD* deletion mutant AhD14, and the lipase activity and aerolysin concentration of the culture supernatant, relative to a strain expressing wild-type ExeD, were determined. All of the mutant strains showed significantly reduced levels of lipase secretion (Fig. 3). The concentration of aerolysin in the wild-type culture supernatant was 152 ng/mL, whereas the aerolysin concentration of the mutant culture supernatants was 34 ng/mL for the VA183 mutant, and 1 ng/mL for all of the other mutants tested. Assembly of the secretin multimer in the two-codon insertion mutants was determined by separating whole cell samples on a 3–8% gradient SDS PAGE gel and immunoblotting with anti-ExeD serum, as described in the materials and methods. With the exception of the insertion in the N0 subdomain, all of the mutants had significantly decreased amounts of ExeD multimer.

### The N0N1 subdomain of P-ExeD is sufficient for interaction with P-ExeB

**Figure 4:**
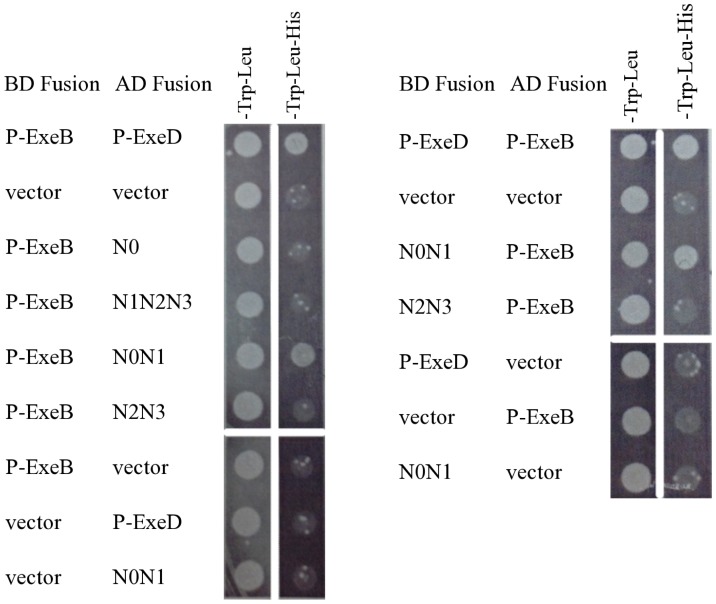
Interaction between the periplasmic domain of ExeB and ExeD subdomain deletion constructs. The P-ExeD deletion constructs, N0, N0N1, N2N3, and N1N2N3 were assayed for interaction with P-ExeB by yeast two-hybrid, as described in Fig. 2.

The results of the two-codon insertion mutagenesis suggested that P-ExeB interacts with the N0 and N1 subdomains of ExeD; therefore, we made deletion constructs containing the subdomains N0, N1N2N3, N0N1, or N2N3 and assayed their ability to interact with P-ExeB by yeast two-hybrid as described above. The BD and AD protein fusions to the N0N1 subdomain of P-ExeD were sufficient to allow growth of yeast on defined medium lacking trp, leu, and his (Fig. 4). Strains containing the N0, N1N2N3, and N2N3 fusions were not able to grow on media without histidine indicating these fragments were unable to interact with P-ExeB.

### In vivo quantification of binding of P-ExeB and P-ExeD

Attempts to measure the P-ExeB-P-ExeD interactions by β-galatosidase assay with the yeast two-hybrid system using strains J694A and Y190 were unsuccessful due to high background activity and low signal strength. Therefore, a bacterial two-hybrid system was used in order to more precisely compare the relative binding when the complete periplasmic domain of ExeD was involved versus its deletion derivatives. In the system employed, interacting pairs drive the expression of the reporter genes *lacZ* and *bla* in *E. coli*. Co-transformants were assayed for the minimum inhibitory concentration (MIC) for carbenicillin and for β-galactosidase activity. All MIC experiments were repeated with a minimum of three independent cultures, and data reported is from one representative experiment. For the MIC assays, a range of 0 to 12.8 mg•mL^−1^ carbenicillin was tested. The vector-vector control strain did not grow at carbenicillin concentrations above 0.1 mg•mL^−1^. The MIC of the positive control, P-ExeB-P-ExeD, and P-ExeD-P-ExeB fusions were 12.8 mg•mL^−1^, 6.4 mg•mL^−1^, and 1.6 mg•mL^−1^, respectively.

**Figure 5:**
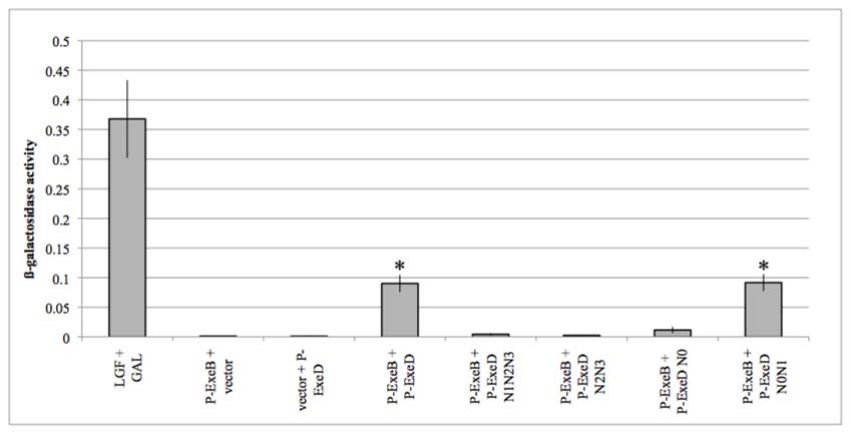
Quantification of the interaction between P-ExeB and P-ExeD or the P-ExeD deletion fragments by bacterial two-hybrid analysis. β-galactosidase activity (ΔOD_420_ per min/(OD_600_×0.1×10)) of *E. coli* co-transformants containing protein fusions to ExeB, ExeD and ExeD deletion constructs. LGF and GAL were used as the positive control. Data presented are the average β-galactosidase activity of three independent cell cultures. Error bars indicate the standard deviation. An asterisk indicates a statistically significant difference in β-galactosidase activity compared to the vector control (P-value <0.001, Student's t-test).

The β-galactosidase activity of the co-transformants was also determined in triplicate. Cells co-expressing the P-ExeB and P-ExeD fusion proteins from plasmids pBT and pTRG, respectively, had β-galactosidase activity that was > 11-fold higher than the activity of cells expressing the empty vectors (Fig. 5). Co-transformants with P-ExeD and P-ExeB in the opposite orientation did not have β-galactosidase activity above the basal level (data not shown). The λcI-ExeD fusion protein expressed from plasmid pBT was not detectable by Western blot, suggesting that the lack of β-galactosidase activity in these cells is due to instability of the λcI-ExeD fusion protein (Fig. S1).

Bacterial two-hybrid analysis was also used to analyze the interaction between P-ExeB and the P-ExeD subdomain deletion mutants (Fig. 5). Western blot analysis confirmed that all of the P-ExeD fragments, except for the N0 subdomain fragment, were stably expressed (Fig. S1). Cells co-expressing the P-ExeB and P-ExeDN0N1 fusion proteins had the same level of β-galactosidase activity as cells co-expressing P-ExeB and the full-length P-ExeD fusion proteins. *E. coli* co-expressing the P-ExeB fusion with the other P-ExeD deletion constructs did not have β-galactosidase activity above background levels, suggesting that these subdomain fragments do not interact with P-ExeB. Collectively the results of the bacterial two-hybrid assays confirm the interaction between the periplasmic domains of ExeB and ExeD. The results also confirm that the N0N1 sub-domains are sufficient for the interaction between P-ExeD and P-ExeB.

### Co-purification of P-ExeB and P-ExeD

**Figure 6:**
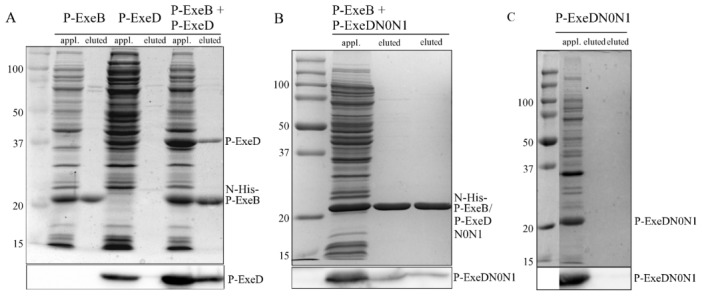
Co-purification of P-ExeD and P-ExeDN0N1 with N-His-P-ExeB. Cell lysates were applied to a Ni affinity chromatography column and eluted with 0.5(upper panel) or immunoblotted with α-ExeD serum (lower panel). Cell lysates from *E. coli* expressing either N-His tagged P-ExeB, or P-ExeD are also shown. Cell lysates from *E. coli* co-expressing untagged P-ExeD and N-His tagged P-ExeB (A), untagged P-ExeDN0N1 and N-His tagged P-ExeB (B) or expressing untagged P-ExeDN0N1 alone (C) were purified and analyzed as described above. The P-ExeDN0N1 and P-ExeB fragments have similar sizes, therefore, in panel B the P-ExeDN0N1 fragment can only be distinguished in the immunoblot.

Co-purification was used to further validate the interaction between P-ExeB and P-ExeD. A pET30a construct containing N-His tagged P-ExeB and P-ExeD constructs in pCDFDuet-1 were co-expressed in *E. coli* cells. Cell lysates were applied to a Ni affinity chromatography column and fractions were analyzed by SDS PAGE or immunoblotting, as specified in the materials and methods. We observed that P-ExeD co-purified with N-His-P-ExeB when lysate from cells co-expressing both proteins was applied to the column and eluted with imidazole (Fig. 6A); however, P-ExeD did not appear in the eluted fractions when lysate from cells expressing only P-ExeD was applied.

A similar approach was used to validate the interaction between P-ExeB and the N0N1 subdomains of P-ExeD that were identified by the yeast and bacterial two-hybrid analysis. The P-ExeDN0N1 fragment co-purified with N-His-P-ExeB when cell lysate from *E. coli* co-expressing both proteins was applied to the Ni affinity chromatography column (Fig. 6B). No detectable P-ExeDN0N1 was observed in the eluted fractions when lysate from cells expressing it alone was applied (Fig. 6C).

### In vitro quantification of binding affinity by rotational anisotropy

**Figure 7:**
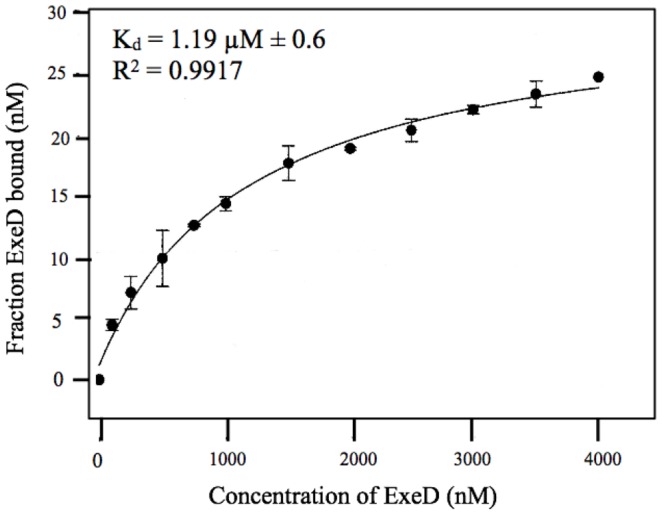
The binding affinity of F-labeled P-ExeB and P-ExeD was measured *in vitro* by rotational anisotropy. Reaction mixtures (50 µL) contained F-labeled P-ExeB (50 nM), RT buffer, and a titration of P-ExeD (0–4000 nM). Samples were excited with vertically polarized light at 495 nm (6-nm band pass) and vertical and horizontal emissions were measured at 520 nm (6-nm band pass). Data collection and anisotropy calculations were performed at 21°C on a QuantaMaster QM-4 spectrofluorometer (Photon Technology International) with a dual emission channel. Data presented are the average (±SD) of at least three independent trials.

Rotational anisotropy was used to determine the dissociation constant (K_d_) for binding of the purified periplasmic domains of ExeB and ExeD *in vitro*. Titration of P-ExeD with fluorescein-EX labeled P-ExeB resulted in data that were best fit to a hyperbolic curve by non-linear regression (R^2^ 0.9917), suggesting non-cooperative binding. The apparent K_d_ of the interaction was 1.19±0.16 µM (Fig. 7). Extensive degradation of the N-His-tagged deletion fragments of P-ExeD was observed during their purification and attempts to determine their binding affinity for the fluorescein-EX labeled P-ExeB were unsuccessful, likely due to mis-folding of the truncated fragments *in vitro*.

### Model building

**Figure 8:**
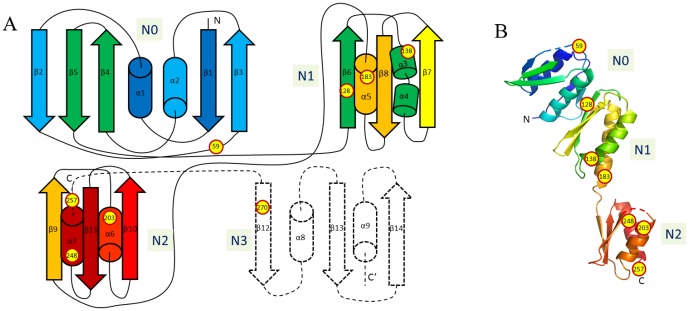
Topology (A) and three dimensional model (B) of the periplasmic domain of ExeD. The position of two amino acid insertion mutations are shown as yellow circles. The dashed line indicates topology of subdomain N3 for which there is no structure available.

We used the crystal structure of GspD from ETEC to perform *in silico* prediction analysis of the effect of the two codon insertions on the tertiary structure of ExeD [13], [36] (PDB code, 3EZJ). Amino acid Arg59 is located in the loop between strands β3 and β4 and should not disrupt folding of the protein (Figure 8). Amino acid Arg128 is located in the β6 strand and it is embedded inside of the molecule. Amino acid Arg138 is located in the α5 helix and is exposed to the solvent. Amino acid Arg183 is located at the beginning of the helix α3 and is partially exposed to the solvent. Amino acid Gly203 is part of the α6b helix and is facing the solvent. Residue Ala248 is located at the beginning of helix α7 and is also facing the solvent. Residue Arg257 is located just after the helix α7 and also faces the solvent. Residue Arg270 is not included in the 3d model due to lack of sequence similarity to known strctures, however from the secondary structure prediction it should be part of strand β12.

## Discussion

In *Aeromonas hydrophila*, assembly of the T2SS secretin, ExeD, requires the inner membrane protein complex ExeAB [16]. In this study we used yeast and bacterial two hybrid analysis, co-purification, and rotational anisotropy to identify and quantify an interaction between the periplasmic domains of ExeB and ExeD. These observations address two important questions that have previously precluded formation of a model for the role of the ExeAB complex in secretin assembly: is the ExeAB complex directly or indirectly involved in secretin assembly, and what is the role of ExeB within the complex? We have previously shown that ExeA binds peptidoglycan and forms a complex with ExeB, both of which are required for assembly of the ExeD secretin in the outer membrane [21]–[23]. This study provides strong evidence that the N0N1 subdomains of ExeD interact directly with this complex through binding with ExeB. Furthermore, our data suggest that ExeB acts as a scaffold protein whose main function is to bring together the proteins ExeA and ExeD in a tri-molecular complex. Collectively, these data support a model for secretin assembly in *Aeromonas hydrophila* in which the ExeAB complex multimerizes in association with peptidoglycan and ExeB acts as a scaffold for assembly by interacting directly with both PG-ExeA and the N0N1 subdomain of ExeD.

Yeast two hybrid analysis was used to assess potential interactions between ExeD and other components of the T2SS in *A. hydrophila*. We identified interactions between ExeD and ExeB, ExeC, and ExeD, respectively. Similar studies have used the yeast two hybrid system to identify protein-protein interactions between the T2SS components of the Out system in *Erwinia chrysanthemi* [7], [34], however, similar OutD-OutD or OutD-OutC interactions were not detected, and OutB was not included in the analysis [34]. Several of the interactions we detected were only present in one orientation, suggesting that these interactions may be sensitive to directionality. The yeast two hybrid vectors pGBT9 and pGAD424 that were used in the study have promoters that are constitutively expressed at low levels in yeast. The fusion proteins were not detectable by Western blot with either GAL4 antibodies, or Exe-specific antibodies. Therefore, it is also possible that the observed negative interactions are due to instability of the fusion proteins. To compensate for these limitations of the yeast two hybrid method, we confirmed the interaction data with additional methods, including bacterial two-hybrid analysis, co-purification and rotational anisotropy.

The periplasmic domain of ExeD is comprised of four subdomains designated N0, N1, N2 and N3 [13]. We used two codon insertion mutagenesis and yeast two hybrid analysis to map the subdomain in P-ExeD that was responsible for interaction with P-ExeB. The insertion in the N0 subdomain (59IR) completely disrupted the P-ExeB-P-ExeD yeast two-hybrid interaction. The P-ExeD-P-ExeD interaction was not disrupted by the 59IR insertion, suggesting that this mutant proteins was stably produced *in vivo*. P-ExeB was able to interact at least partially with all three mutants with insertions in the N1 subdomain (128IR, 138IR, and 183IR), but only in one orientation. In addition, these mutants were unable to secrete the T2SS substrates lipase and aerolysin, and with the exception of 59IR were deficient for secretin assembly. We used the structure of GspD from ETEC to perform in silico prediction analysis of the effect of the two codon insertions on the tertiary structure of ExeD [13], [36]. Our model is based on GspD from enterotoxigenic *E. coli* (PDB; 3EZJ), which was used as the basis for manual modelling using COOT supported by BLAST sequence alignment [13], [37], [38]. The structural modeling (Figure 8) suggested that the 128IR mutation would result in general disruption of N-domain folding, whereas the 59IR mutation would most likely result in only modest changes to the surrounding structure (Figure 8). During formation the secretin N0-N0 interface buries 1100 Å^2^ solvent accessible surface area which is composed of helix α2, strand β2 and the β2-β3 loop, residues from strand β5 and the loop between β4 and β5 [15]. This interface contains only one inserted mutation, after Arg59, located in the β2-β3 loop, which could potentially disrupt the dodecameric helix of N0 [15]. All other insertions are located in the secondary structure elements, but face the solvent. While it is difficult to assess the effect of two amino acid insertions because they can be very disruptive to protein structure, there is a reasonable chance that the mutant ExeD would fold properly. These predictions are supported by the observed effects of the two codon mutations in that the 128IR mutation caused a larger decrease in assembled secretin, than the 59IR, 138IR, and 183IR mutations did.

The 59IR mutation did not affect multimerization of ExeD, and the secretin was localized to the outer membrane (data not shown). A limitation of the yeast 2-hybrid technique is that weak interactions may not be observable above the background level of growth. Therefore, multimerization of ExeD in the 59IR mutant could be due to a weak interaction *in vivo* that was below the limit of detection of the yeast two hyrid assay. To test this hypothesis we used bacterial two hybrid assays to test the interaction between P-ExeB and P-ExeD with the IR59 mutation, and found that cells co-expressing these proteins had approximately 30% of the β-galactosidase activity of the wild-type ExeD (data not shown), suggesting that there is a very weak interacton between these two proteins. It is also possible that structural changes caused by the 59IR mutation allows for ExeAB-independent assembly of the secretin. Notably, while the 59IR mutant was able to assemble ExeD into multimers, there was no observable secretion of lipase or aerolysin. Secretins from other homologous T2SSs in *V. cholera, D. dadantii*, and *P. aeruginosa* have been shown to interact with GspC [31]–[33]. Specifically both the N0 and the N2/N3 subdomains of GspD have been shown to interact with the homologous region of GspC [31]–[33]. We also observed an interaction in the yeast two-hybrid studies between ExeC and ExeD in *A. hydrophila*, although it appeared much weaker than the ExeB-ExeD interaction. In addition, the N0N1 subdomains of the secretin XcpQ from *Pseudomonas aeruginosa* has been shown to interact with substrates of the T2SS, including lipase [35]. Therefore, the nonsecretory phenotype of the 59IR mutant could be due to disruption of other interactions between the N0 subdomain of the secretin and either ExeC or the substrate.

The yeast two hybrid fusion constructs with the two codon insertion mutations in P-ExeD were also used to map the P-ExeD-P-ExeD interaction. All of the mutations, except for 59IR, and 128IR, disrupted the interaction, suggesting that there are important binding sites in the N1 and N2 subdomains that contribute to secretin assembly. Mutations that affected the P-ExeD-P-ExeD interaction also decreased or completely disrupted the multimerization of secretin monomers, as determined by immunoblot analysis. In addition, these mutations negatively affected secretion of the substrates lipase and aerolysin.

Truncated derivatives of the ExeD subdomains were used to confirm the results of the two codon insertion mutagenesis and further refine our determination of the ExeB binding site within ExeD, in this case by demonstrating retention of the interaction. Yeast and bacterial two hybrid assays, and co-purification analysis demonstrated that the N0N1 subdomain is sufficient for binding with P-ExeB. A fragment containing the N1-N3 subdomains did not interact with P-ExeB, suggesting that N1 alone is not sufficient for interaction. The N0 fragment was not detectable by Western blot, suggesting that it may be unstable. Therefore, it was not possible to demonstrate conclusively whether or not N0 is sufficient for interaction with P-ExeB. In addition, attempts to purify and determine the binding affinity of the P-ExeD deletion fragments by rotational anisotropy were unsuccessful, probably due to instability or misfolding of the truncated fragments. These results are consistent with those of Condemine & Shevchik, who also reported that truncated derivatives of the ExeD homolog, OutD in *Erwinia chrysanthemi* were inherently unstable [39].

Structural analysis of the N0N1N2 subdomains of GspD from *E. coli* determined that the N0 subdomain contains similarities to the TonB dependent receptor protein FvpA from *Pseudomonas aeruginosa*. This finding led the authors to speculate that the β2 strand of the N0 subdomain of ExeD may interact with a β strand from a substrate protein, or another T2SS component [13]. More recently, Korotkov *et al*. [15] have provided evidence that the β2 strand is involved in an N0-N0 interface within the assembled secretin. ExeB is 39% similar to TonB and the proteins share a proline enriched region and have similar residue conservation profiles [19]. These findings lead us to hypothesize that ExeB may also interact with the β2 strand of the N0 subdomain of ExeD by a mechanism similar to the interaction between TonB and TonB dependent receptors such as FvpA. A similar hypothesis has been proposed for GspC [13], which has been shown to interact with several sites in GspD, including the N0 subdomain in *D. dadantii* [32] and *V. cholerae* [33]. We also detected a possible weak interaction between P-ExeD and P-ExeC. The P-ExeB interaction is likely only required for assembly of the secretin, whereas the ExeC interaction is not required for assembly, but is important for the secretion function of the assembled secretin [33]. Several of the GspC and GspD interactions in *D. dadantii* were found to have a transient nature [32]. The close proximity of the putative P-ExeB and P-ExeC interaction sites within ExeD, and the fact that they interact relatively weakly, suggests that the interaction between P-ExeB and P-ExeD may also be transient interactions. The transient nature of these interactions, and the fact that the functions of ExeB and ExeC occur during different stages of the secretion process suggest that the ExeB-ExeD and-ExeC-ExeD interactions may occur sequentially, rather than simultaneously.

## Supporting Information

Figure S1 Stability of the P-ExeD fragments used for bacterial two-hybrid analysis. Cell extracts of *E. coli* co-expressing P-ExeB and P-ExeD from plasmids pBT or pTRG were separated by SDS-PAGE and analyzed by Western blotting with detection by anti-ExeD. The immunoblot was overdeveloped so that even small amounts of the pTRGExeDN0 fragment would have been observed if present. The approximate expected sizes of the ExeD fusion protein fragments are: pBTExeD, 61.5 kDa; pTRGExeD, 62.5 kDa; pTRGExeDN1-N3, 51.8 kDa; pTRGExeDN2N3, 43 kDa; pTRGExeN0; 37 kDa; pTRGExeDN0N1, 49.5 kDa.
(EPS)
